# The Quack Doctor’s Own Methods

**Published:** 1893-04

**Authors:** 


### THE QUACK DOCTOR'S OWN METHODS.

For ways that are dark and tricks that are vain the quack doctor may be as peculiar as the heathen Chinee. He is not slow to take advantage of every "nigh cut" to practice, over the broad and easy road of deception and lying. This is a matter of never-failing interest in Georgia, and especially in Atlanta; for down here the quacks are like the poor—always with us. It gives us pleasure to expose one of these whenever he comes in our way. It appears that on February 28th, the Atchison (Kansas) *Daily Globe* published the following :

Dr. Blair, who was recently offered the position of chief surgeon of Grady hospital at Atlanta, Ga., and the Chair of Surgery of the Atlanta Medical College, has declined the offer, and will carry out his original intention of remaining in Atchison. The people of Atchison will learn of his decision with a great deal of pleasure.

A similar item was also published in another issue of the same paper.

In a few days the following letter was received in Atlanta from a prominent surgeon in Atchison :

ATCHISON, KANSAS, March 8th, 1893.

*To the Surgeon in Charge of Grady Hospital, Atlanta, Ga.:*

SIR—Your attention is respectfully drawn to the attached clippings from *The Atchison Daily Globe* relative to work in your hospital. This fellow (Dr. Blair) is such a blatant advertiser that some of the conservative element would like to hear from "the other side" in this matter, hence I take the liberty of writing you. There are so many eminent surgeons in the South that it seems quite unusual that Atlanta should come to Kansas to secure a surgeon to take charge of her principal hospital. As all do not believe all that appears in the newspapers we take this means of giving "the other fellow a chance to tell his story." We would like to hear from you in the matter.

Yours truly, —. —. ———.

A committee of the medical staff of the Grady Hospital then wrote:

ATLANTA, GA., March 18th, 1893.

*Editor Atchison Daily Globe:*

DEAR SIR—Two incorrect statements have lately appeared in your paper, and the undersigned were appointed a committee by the medical board of the Grady Hospital to correct the same, and beg that you will give the correction an equal prominence in the *Globe.*

The facts of the case are these: Dr. E. G. Blair was offered the position of house surgeon and physician on the staff of the hospital, subordinate to the regular attending physicians and surgeons; in that position he would do nothing but carry out their orders and act as their assistant in surgical operations. There is no such position as chief surgeon to the hospital, the position offered him being that of *interne.* As such he would reside in the hospital, and receive a salary of $50.00 per month for the term of twelve months only. The Grady Hospital is a new institution, and it was thought advisable to secure the services of some young man who had just gone through a hospital training and was familiar with the work.

We are informed by Dr. W. S. Kendrick, Proctor of the Atlanta Medical College, that Dr. Blair's name was not thought of in connection with any position in the college, as no vacancy existed.

Signed, VIRGIL O. HARDON, M. D.,
WM. S. ARMSTRONG, M. D.,
HUNTER P. COOPER, M. D., Committee.

This man, we may remark, had had excellent advantages, including a thorough hospital service, and he could doubtless have made his mark in the legitimate profession had he been willing to work and bide his time. With some apologies to Scripture, "he who maketh haste to get (practice) shall not be innocent."

---

COL. ELLIOTT. F SHEPARD, the fire-eating editor of the New York *Mail and Express*, died under ether March 24th. Drs. Chas. McBurney and J. W. McLane were to make an exploration of his bladder for stone, but before the patient was thoroughly anæsthetized his respiration and pulse began to fail and he never recovered. Tracheotomy was done, a rubber tube passed into the trachea, oxygen was administered, and other methods of resuscitation resorted to without success, and the patient died four hours after the administration of ether began. The doctors believed that Col. Shepard "died of sudden œdema and congestion of the lungs, following the administration of ether, but primarily due to some cause unknown to us."

---

PHYSICIANS who will attend the World's Fair will be cordially welcomed at the headquarters of the *Chicago Medical Bulletin*, 1212 Masonic Temple. Information and assistance will be cheerfully rendered by the editor, Dr. Kauffmann. The leading medical journals of the country will also be kept on file for the visiting doctors.

